# High-Resolution Genotyping of Wild Barley Introgression Lines and Fine-Mapping of the Threshability Locus *thresh-1* Using the Illumina GoldenGate Assay

**DOI:** 10.1534/g3.111.000182

**Published:** 2011-08-01

**Authors:** Inga Schmalenbach, Timothy J. March, Thomas Bringezu, Robbie Waugh, Klaus Pillen

**Affiliations:** * Max Planck Institute for Plant Breeding Research, Department of Plant Breeding and Genetics, 50829 Cologne, Germany,; † Martin-Luther-University Halle-Wittenberg, Institute of Agricultural and Nutritional Sciences, Chair of Plant Breeding, 06120 Halle/Saale, Germany, and; ‡ Scottish Crop Research Institute, Invergowrie, Dundee DD2 5DA, United Kingdom

**Keywords:** wild barley introgression lines, Illumina GoldenGate assay, high-resolution mapping populations, threshability locus *thresh-1*

## Abstract

Genetically well-characterized mapping populations are a key tool for rapid and precise localization of quantitative trait loci (QTL) and subsequent identification of the underlying genes. In this study, a set of 73 introgression lines (S42ILs) originating from a cross between the spring barley cultivar Scarlett (*Hordeum vulgare* ssp. *vulgare*) and the wild barley accession ISR42-8 (*H. v*. ssp. *spontaneum*) was subjected to high-resolution genotyping with an Illumina 1536-SNP array. The array enabled a precise localization of the wild barley introgressions in the elite barley background. Based on 636 informative SNPs, the S42IL set represents 87.3% of the wild barley genome, where each line contains on average 3.3% of the donor genome. Furthermore, segregating high-resolution mapping populations (S42IL-HRs) were developed for 70 S42ILs in order to facilitate QTL fine-mapping and cloning. As a case study, we used the developed genetic resources to rapidly identify and fine-map the novel locus *thresh-1* on chromosome 1H that controls grain threshability. Here, the recessive wild barley allele confers a difficult to thresh phenotype, suggesting that *thresh-1* played an important role during barley domestication. Using a S42IL-HR population, *thresh-1* was fine-mapped within a 4.3cM interval that was predicted to contain candidate genes involved in regulation of plant cell wall composition. The set of wild barley introgression lines and derived high-resolution populations are ideal tools to speed up the process of mapping and further dissecting QTL, which ultimately clears the way for isolating the genes behind QTL effects.

As demonstrated in various crop species, introgression lines (ILs) are a valuable genetic resource for the identification of QTL for important agronomic traits (*e.g.*, Eshed and Zamir 1995; Faris and Gill 2002; Liu *et al.* 2006; Mei *et al.* 2006; Simons *et al.* 2006; Uauy *et al.* 2006; Szalma *et al.* 2007; Falke *et al.* 2008; Fu *et al.* 2009). An IL set represents the genome of a donor parent through single lines each carrying one or few introgressed donor segments in the same genetic background of the recurrent parent. This is achieved by several rounds of backcrossing to the recurrent parent followed by marker-assisted selection (Zamir 2001). Compared with traditional mapping populations such as recombinant inbred lines (RILs), ILs offer increased statistical power to detect small QTL effects due to the removal of confounding segregating alleles at background genomic regions (Law 1966; Keurentjes *et al.* 2007). As a result, complex traits, controlled by several unlinked genes, can be broken down into simple Mendelian factors (Deng *et al.* 2011). In addition, because only small genetic regions are introgressed, favorable alleles from exotic species can be incorporated, exhibiting no or only a limited number of additional unfavorable effects that might cosegregate as linkage drag. All in all, these factors make ILs a useful base to embark on fine-mapping and cloning of important QTL.

ILs developed in tomato have been used extensively to map QTL controlling complex quantitative traits including fruit weight, sugar content, and plant size (Alpert and Tanksley 1996; Eshed and Zamir 1995). In barley, a subset of near isogenic lines were developed for fine-mapping a locus controlling leaf rust (Marcel *et al.* 2007). QTL controlling grain weight (Röder *et al.* 2008) and the number of grains per ear (Wang *et al.* 2010a) were fine-mapped in wheat using ILs. Additionally in tomato, segregating populations derived from ILs containing QTL facilitated the cloning of genes controlling fruit size (Frary *et al.* 2000) and fruit sugar content (Fridman *et al.* 2004).

A dense genetic map which allows for localizing the introgressed segments with high resolution is crucial for the selection of ILs containing only a small portion of the introgressed genome. So far, the majority of IL sets has been developed and applied for QTL mapping based on a relatively low number of markers that have been generated from anonymous genomic regions (*e.g.*, simple sequence repeats, restriction fragment length polymorphisms). Currently, gene-based molecular markers originating from cDNAs/ESTs (expressed sequenced tags) have gained importance in plant genetics and genomics-assisted breeding (*e.g.*, Kota *et al.* 2008; Deleu *et al.* 2009; Varshney *et al.* 2009; Chin *et al.* 2010). Here, EST–single nucleotide polymorphisms (SNPs) are especially useful due to their high abundance and adaptability to high-throughput, low-cost genotyping assays. Markers derived from ESTs allow for detecting polymorphisms within protein-coding transcribed genes and thus may facilitate gene isolation via map-based cloning or comparative genomics (Stein *et al.* 2007; Hackauf *et al.* 2009; Muchero *et al.* 2011).

In barley (*Hordeum vulgare*), Close *et al.* (2009) identified a significant number of genic SNPs from ESTs and sequenced PCR amplicons and used them to develop two Illumina barley oligo pool assays (BOPA1 and BOPA2), each enabling the simultaneous genotyping of 1536 SNPs. Furthermore, a barley consensus genetic map comprising 2943 SNPs has been created from linkage maps of four reference populations (Close *et al.* 2009). The advantage of these resources has been proven by diverse association mapping studies (Rostoks *et al.* 2006; Cockram *et al.* 2010; Comadran *et al.* 2011; Lorenz *et al.* 2010). So far, two collections of backcross-derived barley lines have been developed and characterized using the Illumina genotyping arrays and the above described consensus map. Druka *et al.* (2011) constructed a set of near isogenic lines carrying mutant alleles for most of the morphological and developmental variation in barley. Sato and Takeda (2009) reported on the characterization of a set of recombinant chromosome substitution lines, each containing a small portion of a wild barley accession introgressed into the genetic background of an elite parent. Both populations are proposed as valuable resources for identifying genes underlying simple and quantitative traits.

Such populations might also be helpful to shed further light on genes which were selected during domestication. In barley domestication-related genes like brittleness, spike row-type, flowering time control under photoperiod and vernalization signals are already mapped or cloned (Azhacuvel *et al.* 2006; Komatsuda *et al.* 2007; Ramsay *et al.* 2011; Turner *et al.* 2005; Yan *et al.* 2004, 2005, 2006). Further genes that control important domestication traits like grain size, grain yield or threshability of grains are still awaiting molecular discovery.

In the present study, we aimed to characterize the previously selected barley S42IL population, originating from the cross Scarlett (*H. vulgare ssp. vulgare*, hereafter abbreviated *Hv*) × ISR42-8 (*H. vulgare ssp. spontaneum*, hereafter abbreviated *Hsp*) (Schmalenbach *et al.* 2008) by genotyping with the Illumina BOPA1 array (Close *et al.* 2009). In addition, we intended to further develop the S42ILs into a resource for rapid identification, fine-mapping, and positional cloning of QTL. Besides a dense genetic map, the major requirement for these applications is the development of large populations with sufficient recombination events in the target QTL region. In order to increase the recombination events within the target QTL interval, we developed for most introgression lines a high-resolution mapping population (S42IL-HR) segregating within the introgressed region. As a case study, we describe the application of the developed resources to rapidly identify and fine-map a genomic region on chromosome 1H containing the novel locus *thresh-1*, derived from *Hsp*, which controls grain threshability.

## Materials and Methods

### Plant material

**Figure 1:**
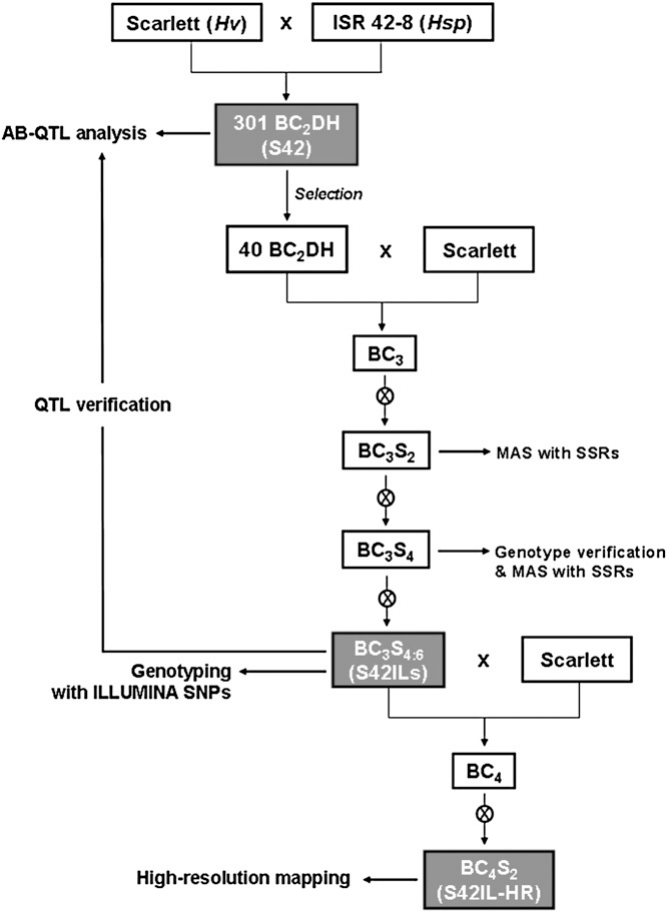
Strategy for developing introgressions lines and high-resolution mapping populations from the wild barley cross Scarlett × ISR42-8. In order to select a set of introgression lines (S42ILs), a strategy combining backcrossing, repeated selfing, and marker-assisted selection (MAS) was applied. In the BC_2_S_4:6_ generation, the S42ILs have been characterized precisely on the genotype level using Illumina SNPs as described in the present study. Additionally, phenotype data were assessed for a subset of 39 S42ILs in order to validate QTL effects detected in the parental population S42 (Schmalenbach *et al.* 2008; Schmalenbach *et al.* 2009; Schmalenbach and Pillen 2009). High-resolution mapping populations (S42IL-HR) were finally developed by backcrossing the S42ILs with the recurrent parent followed by two selfings. They are available for future fine-mapping and cloning of interesting genes.

A set of 73 wild barley introgression lines (hereafter referred to as S42ILs) was subjected to high-throughput genotyping with the Illumina GoldenGate assay. The S42ILs are derived from a cross between the German malting barley cultivar Scarlett and the Israeli wild barley accession ISR 42-8. As reported in Von Korff *et al.* (2004), the initial cross was backcrossed twice to produce the advanced backcross doubled haploid population S42 consisting of 301 BC_2_DH lines. The S42ILs were generated based on 40 lines selected from that population (Von Korff *et al.* 2004). Here, a strategy combining one further round of backcrossing, two to four selfings and marker-assisted selection with simple sequence repeat (SSR) markers was applied (Figure 1). The development of 59 S42ILs (S42ILs-101 to -157, -175, and -176) as well as their initial genetic characterization with altogether 98 SSR markers is described in detail in Schmalenbach *et al.* (2008). Since then, 14 new lines (S42ILs-158 to -164, -166 to -171, and -173) were selected following the same strategy as described above (Figure 1). Based on the initial SSR genotype data, each single line contained one or two chromosomal segments of the exotic parent ISR 42-8 (*Hsp*) within the genetic background of the elite parent Scarlett (*Hv*) (Schmalenbach *et al.* 2008). The genotyping of the 73 S42ILs with the Illumina GoldenGate assay was carried out with BC_3_S_6_ plants which originate from a single BC_3_S_4_ line (S42IL).

To enable fine-mapping and map-based cloning of interesting phenotypes, high-resolution mapping populations derived from the S42ILs were generated through one further round of backcrossing with Scarlett and two consecutive rounds of selfing (Figure 1). For backcrossing, one to five S42IL plants in BC_3_S_6_ were randomly chosen to produce BC_4_S_0_ seeds. For selfings, at least 6 BC_4_S_0_ seeds and, respectively, 100 BC_4_S_1_ seeds were randomly chosen to reduce the risk of selection or drift effects present in the resulting BC_4_S_2_ HR populations.

### Extraction of genomic DNA

For DNA isolation, 30-50 mg leaf material of 2-week-old seedlings grown in the greenhouse was harvested for each S42IL. Per line, leaf material from on average 12 BC_3_S_6_ plants was pooled. After adding 400 µl RLT buffer (Qiagen, Hilden, Germany), the material was homogenized using a TissueLyser bead mill (Qiagen) and extracted using the BioSprint DNA Plant Kit and the BioSprint 96 workstation from Qiagen. Isolated DNA was disolved in distilled water, and, based on agarose gel electrophoresis, DNA concentration of all samples was assessed. If required, samples were concentrated using a Savant SpeedVac concentrator (GMI) to achieve a final concentration of about 100 ng/µl. For Illumina SNP genotyping, a sample volume of 25 µl was provided.

### Genotyping and characterization of the S42ILs with the Illumina GoldenGate assay

DNA samples were submitted to the Southern California Genotyping Consortium (SCGC), Illumina BeadLab at the University of California, Los Angeles (http://scgc.genetics.ucla.edu/) and genotyped with the 1536-SNP barley BOPA1 set (Close *et al.* 2009). Forty-one S42ILs (S42IL-101 to -139, -149, and -150) were genotyped in two technical replications and the remaining 32 lines in one replication. In addition, the parents Scarlett and ISR42-8 were analyzed in four replicates each.

**Figure 2:**
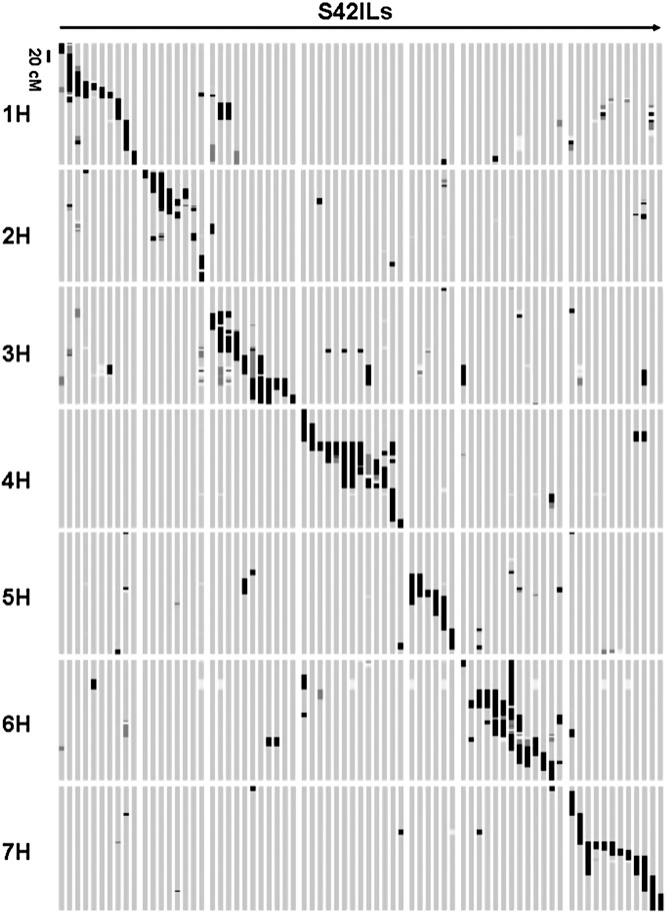
Graphical genotypes of 73 *Hsp* introgression lines (S42ILs). The 73 S42ILs, presented vertically, have been characterized with 636 Illumina SNPs, shown horizontally. The order of the S42ILs is in accordance with Table 1, and the marker order is taken from the consensus map of Close *et al.* (2009). Each S42IL carries one or several homozygous or heterozygous wild barley introgressions (depicted in black and dark gray, respectively) in the genetic background of the elite parent (in light gray). Missing marker data are indicated in white.

Obtained raw data were transformed to genotype calls and subsequently manually supervised to correct for excessive emphasis on heterozygote calls using GenCall software (Illumina, San Diego, CA) at the Close lab (University of California, Riverside, CA). Only the most reliable calls were retained. All SNPs, which had no genotype or map data, were nonpolymorphic between the parents, or showed ambiguous and nonreproducible genotypes, were discarded. The informative SNPs used for characterizing the S42ILs are designated by BOPA1 numbers (Close *et al.* 2009). Detailed information such as the according HarvEST unigene assembly #32 numbers is given in supporting information, Table S1. The genetic order of all markers was taken from the Close *et al.* (2009) consensus map which included 2943 SNP loci. Based on their graphical genotypes, obtained using the Graphical GenoTypes (GGT) software (Van Berloo 1999), the S42ILs where ordered according to the chromosomal positions of their overlapping target introgressions (Figure 2 and Table S1). The size of the target introgression was calculated for each line, where the half-intervals flanking a marker locus were assumed to be of the same genotype. For calculating the portion of *Hsp* genome per S42IL, a total genome size of 1576 cM was assumed, based on Close *et al.* (2009) (see Table S1).

### Phenotyping for grain threshability

**Figure 3:**
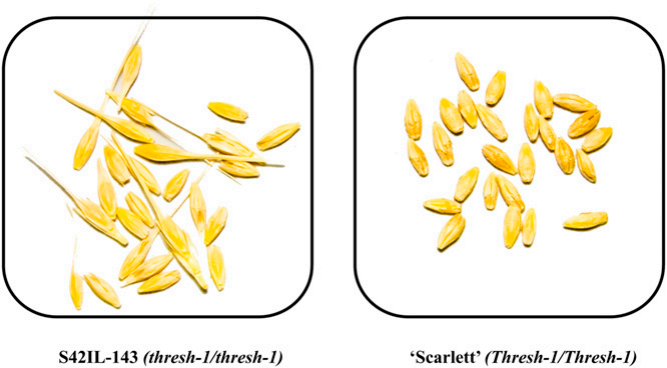
Phenotypes of the *thresh-1* gene after mechanical threshing. Left image: Difficult to thresh grains from line S42IL-143, homozygous for the recessive *Hsp* allele *thresh-1* where parts of the awns and the rachis remain attached to the grain. Right image: Easy to thresh grains from cultivar Scarlett, homozygous for the dominant *Hv* allele *Thresh-1* without remnants from awns and rachis.

To evaluate grain threshability (see Figure 3) a subset of 49 S42ILs (S42IL-101 to -144, -146 to -149, and -153) and Scarlett as the control genotype were evaluated in two independent glasshouse experiments. Each experiment consisted of three completely randomized blocks. Ten plants of each genotype were grown in 1.5 L pots filled with a cultivation substrate containing peat, clay, and NPK fertilizer with 250, 300, and 400 mg/L nitrogen, phosphate, and potassium, respectively, pH 5.8. Plants were grown under 14/10-hr day/night photoperiod with a daily temperature range of 15°C to 22°C. At maturity grain spikes were collected and threshed using a rotating home-made threshing drum. The rotational speed of the threshing drum and duration of threshing was adjusted so that grains from Scarlett were completely removed from the spike rachis and the awns were detached from the grain. Threshability was scored as a qualitative trait, where a genotype was considered difficult to thresh if parts of the awns and/or the rachis remained attached to the grain after the outlined threshing procedure (Figure 3). Based on the results of this initial experiment, 91 individual seeds from the high resolution mapping population S42IL-143HR were selected depending on their threshability phenotype. The selected HR individuals were grown, along with S42IL-143 and Scarlett as controls, in a second experiment under the same glasshouse conditions as stated above and phenotyped for threshability to enable fine-mapping of the grain threshability locus.

### Genotyping the S42IL-143HR population

For genotyping of the S42IL-143HR population, the Illumina BOPA1 markers were converted to either cleaved amplified polymorphic sequence (CAPS) or pyrosequencing markers as described previously (Wang *et al.* 2010b). Primer information is detailed in Table S2. In total nine BOPA1 markers were converted to genotype *Hsp* introgressions on chromosomes 1H, 5H, 6H, and 7H. In addition, the SSR marker HvABAIP, which was originally used to select the line S42IL-143 (Schmalenbach *et al.* 2008), along with the CAPS marker HvFT3 previously shown to map within the 1H introgression of S42IL-143 (Wang *et al.* 2010b) were also used for genotyping. Genetic distances between the markers were calculated with the Kosambi mapping function in JoinMap v.3.0 (Kyazma B.V, Wageningen, The Netherlands) and graphical genotypes were visualized with the GGT software (Van Berloo 1999).

### Identification of genes in the *thresh-1* region

The previously published virtual gene order of barley chromosome 1H (Mayer *et al.* 2009) was used to predict the genes located in the *thresh-1* region. The SSR marker HvABAIP (GenBank accession X13498) was positioned into the chromosome 1H gene order scaffold by performing a blastx search against the rice genome sequence to identify the orthologous gene position in rice (http://blast.ncbi.nlm.nih.gov).

## Results

### Genetic characterization of the S42ILs

A set of 73 wild barley introgression lines was genotyped with high resolution using the Illumina GoldenGate assay. Out of 1536 BOPA1 SNPs, 1148 markers gave useful genotype information in the S42IL set. Of these, a total of 636 SNPs (55.4%) were polymorphic between Scarlett and ISR42-8 and were finally used for characterizing the S42ILs. The average marker density varied from 1.9 cM for chromosome 2H (228 cM/120 SNPs) to 3.1 cM for chromosome 7H (230 cM/75 SNPs). Overall, 87.3% of the *Hsp* genome (1376 out of 1576 cM) is represented by, in most cases overlapping, target *Hsp* introgressions in the *Hv* genetic background (Figure 2). Wild barley chromosomes 1H, 4H, 6H, and 7H are completely represented, whereas the lowest genome coverage was estimated for 5H (63.3%). The biggest gaps where no lines are developed yet were detected on top of chromosomes 5H and 3H with 98.4 cM and 49.6 cM, respectively.

A number of lines carry a second nontarget *Hsp* segment that covers a chromosomal region not represented by primary introgressions in the complete set of lines. An example is S42IL-114 possessing the target introgression on chromosome 3H plus an additional segment on chromosome 5H, which has a size of 14.4 cM (see Table S1). Taking into account these additional introgressions, the total *Hsp* genome coverage of the S42IL set increases to 89.5%.

As shown in Table 1, 20 S42ILs carry a single introgression (*i.e.*, only the target one), 22 lines contain one additional homozygous or heterozygous *Hsp* segment, and 31 lines exhibit two to four secondary introgressions. Three target segments are represented by two lines simultaneously (*i.e.*, S42IL-118 and -120, and -119 and -120 on chromosome 4H, and -149 and -152 on chromosome 6H). The target introgressions possess an average size of 42.9 cM, ranging from 5.7 cM in S42IL-110 to 109.7 cM in S42IL-114 (Table 1). Taking into account all homozygous introgressions, on average 3.3% of the wild barley genome is represented per line, varying from 0.8% in S42IL-170 to 8.4% represented by four independent introgressions in S42IL-156. Furthermore, 38 lines exhibited heterozygosity at one or more SNP loci. A core set of 32 S42ILs that represents the minimum number of lines required to cover the *Hsp* genome was selected (Table 1). Here, preferably lines with overlapping introgressions were picked to ensure a maximum coverage of the donor genome. The remaining 41 S42ILs are partial duplicates with shorter introgressions that can be useful for fine-mapping of markers and QTL.

**Table 1:** Genetic characteristics of 73 *Hsp* introgression lines (S42ILs) based on genotyping with 636 Illumina SNPs

S42IL*^a^*	Chromosome*^b^*	Position of Start SNP*^c^*	Position of End SNP*^d^*	Size of Target Introgression*^e^*	No. of Additional Introgressions*^f^*	*Hsp* (%)*^g^*	Heterozygous (%)*^h^*	S42IL-HR (g)*^i^*
−101	1H	1.10	13.50	17.7	3	1.1	2.2	715
−102^*^		1.10	98.23	99.2	2	5.2	2.2	673
−103		40.51	89.01	51.1	3	3.0	3.1	968
−157		64.79	90.92	28.6	1	2.3	0.0	i.p.
−104		70.78	78.03	11.9	2	1.9	0.0	703
−105		74.40	90.92	18.5	0	1.2	0.0	293
−158		82.35	90.92	10.5	1	1.9	0.0	i.p.
−141^*^		94.86	127.71	36.3	2	3.0	0.2	504
−143^*^		130.68	173.49	51.8	4	4.2	1.6	1180
−142^*^		188.50	205.07	24.1	0	1.5	0.0	348
−106^*^	2H	22.35	34.31	19.1	0	1.2	0.0	865
−107		34.31	66.78	42.0	1	3.3	0.0	607
−108^*^		34.31	104.81	77.9	1	5.0	0.8	290
−109^*^		63.96	110.84	42.4	0	3.3	0.0	984
−153^*^		108.71	120.83	13.3	3	1.8	0.3	1183
−144		63.96	81.50	21.3	1	1.4	0.0	421
−110^*^		102.66	104.81	5.7	2	1.4	0.0	47
−175^*^		197.39	247.86	52.6	4	3.5	1.8	i.p.
−111^*^	3H	67.01	98.41	34.9	4	3.7	1.9	1062
−154^*^		64.85	144.30	84.8	2	6.9	2.3	i.p.
−155		104.39	144.30	46.7	4	5.5	0.5	i.p.
−112^*^		104.39	161.43	64.3	1	3.8	1.7	1312
−159		154.99	190.87	40.1	1	4.9	0.0	i.p.
−114		138.00	245.49	109.7	3	4.6	3.9	1392
−140^*^		154.99	253.73	101.0	0	6.2	0.0	2096
−115^*^		204.48	255.13	53.7	1	4.3	0.0	791
−160		204.48	221.43	24.8	1	2.5	0.0	i.p.
−113		204.48	239.73	38.3	0	2.4	0.0	396
−161		239.73	253.73	19.1	0	1.2	0.0	i.p.
−116^*^	4H	5.42	47.80	49.1	2	5.3	0.0	724
−117^*^		27.52	64.77	41.2	0	2.6	0.0	529
−145		61.15	64.77	13.8	2	1.6	1.0	1073
−118^*^		61.15	83.58	32.2	1	2.5	0.0	957
−120		61.15	83.58	32.2	0	1.4	0.7	1352
−119		61.15	119.06	69.5	1	4.9	0.0	978
−162		61.15	119.06	69.5	0	4.4	0.0	No
−164		61.15	99.74	49.3	1	3.5	0.0	29
−121		74.11	119.06	50.6	2	3.6	2.1	952
−146^*^		83.58	119.06	43.1	0	1.9	0.6	1322
−166		91.93	119.06	32.0	1	2.5	0.0	No
−123^*^		128.85	172.32	50.5	3	5.5	0.0	1174
−124^*^		171.25	183.54	13.4	2	2.4	0.0	1339
−173^*^	5H	104.73	171.34	74.1	0	4.7	0.0	i.p.
−125		104.73	154.37	56.0	1	3.6	0.5	1095
−147		145.57	154.37	17.3	1	1.1	0.2	i.p.
−126		145.57	200.12	61.8	0	3.9	0.0	400
−176^*^		154.37	234.98	81.8	3	6.3	0.8	No
−127^*^		231.75	276.77	50.0	0	3.2	0.0	1349
−148	6H	3.28	10.73	12.7	2	3.5	0.1	1519
−150		73.90	82.43	13.5	1	1.3	0.0	1167
−152		71.39	82.43	31.2	3	3.5	0.3	i.p.
−149		71.39	82.43	31.2	1	2.4	0.0	1659
−128		71.39	132.23	77.8	1	5.2	0.3	1394
−129		73.90	133.47	63.0	0	3.5	0.5	303
−156^*^		89.78	156.09	72.1	3	8.4	1.5	55
−130		98.66	180.69	83.8	2	4.9	1.1	1250
−131^*^		140.00	180.69	47.2	0	2.3	0.7	1244
−163		137.78	163.56	31.1	2	2.0	0.2	i.p.
−132		160.38	191.46	31.6	0	2.0	0.0	185
−122^*^		180.69	208.13	32.8	3	3.5	1.2	1007
−151		98.66	111.56	16.5	3	2.2	0.7	1419
−133^*^	7H	17.32	51.93	44.0	4	4.9	0.7	302
−134^*^		51.93	107.44	59.1	1	3.7	0.9	1468
−135^*^		101.23	152.29	63.1	0	4.0	0.0	29
−167		101.23	114.58	14.4	1	0.9	0.1	i.p.
−168		101.23	116.68	16.5	2	1.5	1.6	40
−169		101.23	120.92	26.4	2	1.7	0.8	17
−170		114.58	120.92	13.1	0	0.8	0.0	i.p.
−171		116.68	134.43	19.5	1	1.3	0.2	42
−136		134.43	152.29	36.7	2	3.5	0.0	1137
−137^*^		134.43	193.89	66.8	4	8.1	0.2	948
−138^*^		176.37	229.66	65.3	1	4.8	1.1	841
−139		198.70	229.66	31.5	0	2.0	0.0	474
Average				42.9	1.5	3.3	0.6	

^a^ Order of the lines follows chromosomal position of the target introgression and corresponds to the order in Table S1. The 32 lines of the S42IL core set are indicated by asterisks (^*^).

^b^ Chromosomal location of the target introgression, based on Close *et al.* (2009).

^c^ Chromosomal position of the first marker of the target introgression (in cM).

^d^ Chromosomal position of the last marker of the target introgression (in cM).

^e^ Estimated size of the target introgression (in cM).

^f^ Number of *Hsp* segments, additional to the target introgression.

^g^ Percentage of homozygous *Hsp* genome per line, based on a total genome size of 1576 cM.

^h^ Percentage of heterozygous loci per line.

^i^ Grams (g) of seed available for each BC_4_S_2_ population. An HR population consisting of 400 g of seed corresponds to 10,000 segregating BC_4_S_2_ individuals assuming an average thousand grain weight of 40g (Schmalenbach *et al.* 2009). The abbreviation “i.p.” indicates that the HR population is currently in preparation through field multiplication.

### Development of high-resolution mapping populations

After backcrossing the S42ILs with Scarlett and two further rounds of selfing, a library of 70 high-resolution populations (S42IL-HR) in BC_4_S_2_ generation was developed (Table 1). Each HR population includes between 17 and 2096 grams of seed, segregating for markers and genes that are located within the introgression of the original S42IL. Assuming an average thousand grain weight of 40 g (Schmalenbach *et al.* 2009), these values correspond to a range from 425 to 52,400 segregating BC_4_S_2_ individuals per HR population. So far, 42 HR populations consist of at least 400 g of seed (*i.e.*, ≥10,000 individuals). Fourteen HR populations are currently multiplied again in the field, as indicated in Table 1. A first application of a HR population is shown by the following mapping of the *thresh-1* locus.

### Genetic mapping of the thresh-1 locus

From 49 examined S42ILs, only line S42IL-143 was found to show reduced grain threshability compared with the control genotype Scarlett. After mechanical threshing, rachis and awns of line S42IL-143 were only partially removed from the grains. Line S42IL-143 was therefore classified as difficult to thresh (Figure 3). The BOPA1 genotype data showed that S42IL-143 possess a major *Hsp* introgression on the long arm of chromosome 1H (51.8 cM) plus four additional *Hsp* introgressions on chromosomes 5H (3.2 cM and 7.0 cM), 6H (28.9 cM), and 7H (4.7 cM). The additional introgressions on chromosomes 5H, 6H, and 7H were also represented by other S42ILs that did not show the difficult to thresh phenotype, thus it was assumed that the *thresh-1* gene is located within the chromosome 1H introgression.

**Figure 4:**
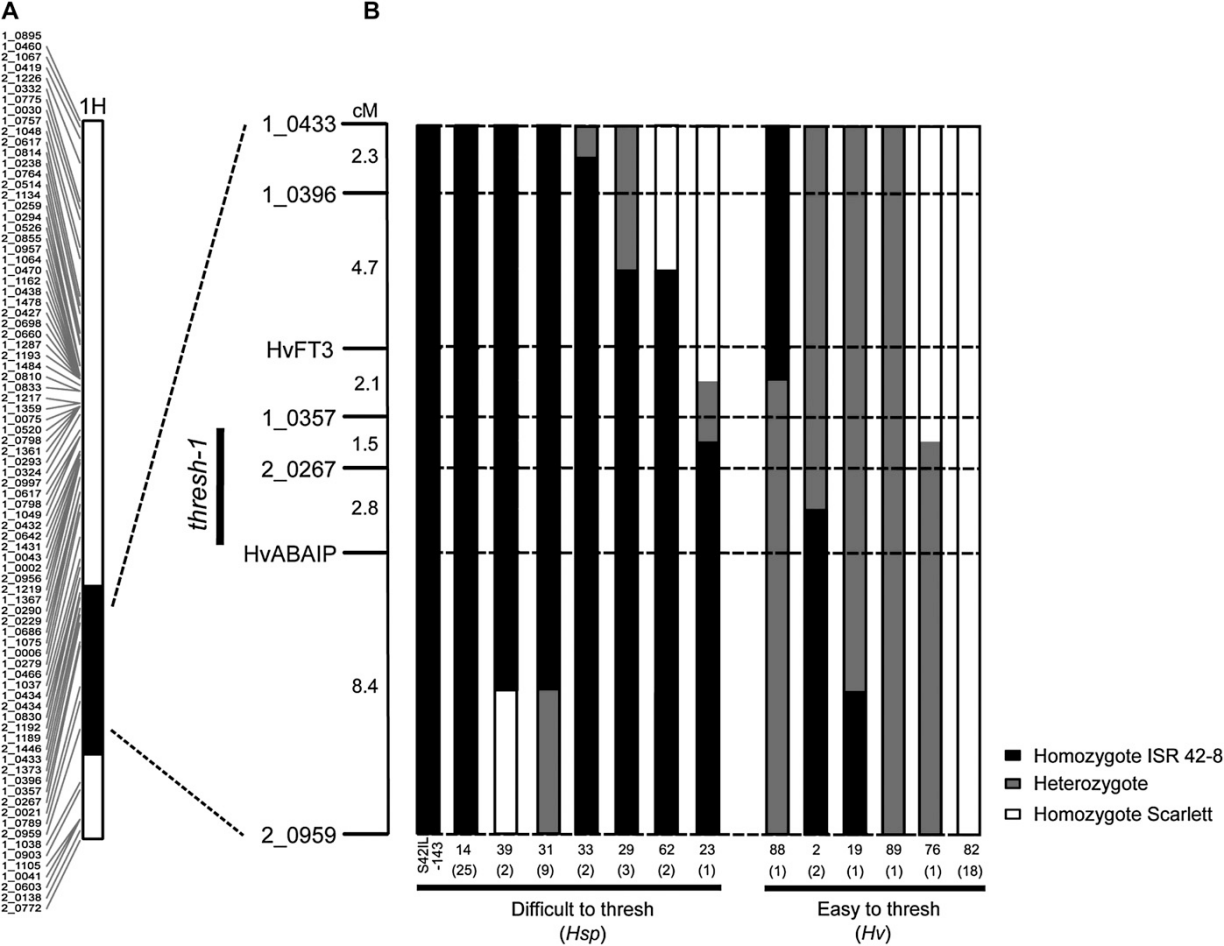
Fine mapping the *thresh-1* locus using the high resolution mapping population S42IL-143HR. (A) Screening 49 S42ILs localized the *thresh-1* gene to an *Hsp* introgression on chromosome 1H, which is present in line S42IL-143. (B) Graphical genotypes showing informative recombinants, identified among 91 BC_4_S_2_ individuals of the S42IL-143HR population, that were used to delimitate the *thresh-1* interval based on their threshability phenotype. Lines with missing or ambiguous genotype data were excluded. The numbers below the graphical genotypes refer to the HR line depicted, while the numbers in brackets refer the total number of individuals identified with the same graphical genotype. Progeny tests from HR lines 2, 19, 88, and 89 proved that the elite (*Hv*) allele *Thresh-1* is dominant over the exotic (*Hsp*) allele *thresh-1*.

The location of *thresh-1* on chromosome 1H was further confirmed by phenotyping threshability of 91 plants of the high-resolution mapping population S42IL-143HR. Simultaneously, the same plants were genotyped with converted BOPA1 markers (Table S2) from all four introgressions of S42IL-143. The genotyping confirmed the presence of the additional *Hsp* introgressions on 5H, 6H, and 7H in S42IL-143. The phenotyping, however, proved that these loci were not genetically linked to the *thresh-1* locus. In contrast, recombinants identified within the 1H *Hsp* introgression allowed to map the *thresh-1* gene within a 4.3-cM interval between markers 1_0357 and HvABAIP (Figure 4). From 91 HR individuals phenotyped, 38 were easy to thresh and 53 were difficult to thresh. However, this segregation ratio was greatly distorted due to the individual selection of seeds, resulting in an overrepresentation of difficult to thresh genotypes. To clarify the mode of inheritance, four HR lines (#2, 19, 88, 89 in Figure 4) that proved to be heterozygous for the *thresh-1* interval between markers 1_0357 and HvABAIP were used for progeny validation. From a total of 88 progeny (*i.e.*, 22 offspring plants from each heterozygous HR plant), 61 were easy to thresh and 27 were difficult to thresh. This was not significantly different from a 3:1 segregation ratio (*x*^2^ = 1.151, *P* = 0.218) confirming that the *Hv* allele is dominant over the *Hsp* allele at the *thresh-1* locus.

### Prediction of genes within the thresh-1 region

The previously published virtual gene order of barley chromosome 1H was used to predict the genes within the *thresh-1* region (Mayer *et al.* 2009). Reciprocal blast searches identified the SSR marker HvABAIP (ABA inducible protein; (Pillen *et al.* 2000), which flanks the bottom of the *thresh-1* locus, to be a putative ortholog of the rice gene Os05g0542500. This allowed anchoring HvABAIP to the chromosome 1H virtual gene order between BOPA1 markers 2_0780 and 2_0921. The BOPA1 marker 2_0267, which flanks the top of the *thresh-1* locus, was already present in the virtual gene order, therefore enabling the *thresh-1* interval to be located. Based on the virtual gene order for this region, which was derived from synteny with the rice and sorghum genomes, the interval containing the *thresh-1* locus was predicted to contain 60 genes (see Table S3). Of particular interest regarding the threshability trait were the identification of cell wall–related genes that encode a cellulose synthase-like family C protein and polygalacturonase.

## Discussion

### Genetic characterization of the S42ILs

The Illumina BOPA1 assay was applied for characterizing a set of 73 *Hsp* introgression lines with high resolution. In total, 636 SNPs out of 1148 (55.4%) revealed polymorphic alleles between the recurrent parent Scarlett and the donor parent ISR42-8. This polymorphism rate is similar to the amount of polymorphic BOPA1 SNPs between the elite barley cultivar Haruna Nijo and the wild barley strain H602 (51%), reported by Sato and Takeda (2009). As described by Close *et al.* (2009), SNP frequency was clearly increased when including H602 in the genotype panel used to develop the BOPA1 assay. Nevertheless, polymorphism rates around 50% between *Hv* and *Hsp* genotypes appear to be relatively low. This might be attributed in part to the SNPs’ origin from coding sequences. An ascertainment bias in favor of *Hv* alleles during the development of the ILLUMINA array might also contributed to the observed polymorphism rate (Moragues *et al.* 2010).

The S42IL set is an immortal genetic resource that represents most of the parental wild barley genome, where each single line contains only a small proportion of the *Hsp* donor genome. Initially, the set was developed and characterized with 98 SSR markers (Schmalenbach *et al.* 2008). Based on the SSR data, each line was assumed to contain only a single homozygous *Hsp* introgression in the genetic background of the elite parent. Genotyping the S42-ILs with the BOPA1 SNPs resulted in an increased mapping precision of the introgressed segments. This, in turn, revealed the existence of additional nontarget introgressions in S42ILs, which had not been detected previously. So far, eight randomly chosen S42ILs were regenotyped with 25 informative pyrosequencing markers in order to verify these secondary introgressions. The latter were derived from original Illumina SNPs (Table S2). The original Illumina assays of these markers had detected 28 *Hsp* introgressions among the eight S42ILs. Through pyrosequencing, the genotype of all 28 *Hsp* introgressions could be verified as homozygous *Hsp* or heterozygous *Hv/Hsp* (data not shown). The complete validation of all tested BOPA1 genotypes demonstrates the high accuracy of the Illumina GoldenGate assay.

Pure S42ILs carrying only the target segments are advantageous as they enable the localization of a genetic effect (*e.g.*, QTL) to a specific chromosomal interval. For this reason, the selection of pure introgression lines from the existing S42IL-HR populations is currently in progress. Nevertheless, S42ILs with overlapping introgressions are also useful for validating and confining the genetic localization of a phenotypic effect as shown in previous S42IL studies on pathogen resistances, yield and its agronomic components, malting quality and flowering time control (Schmalenbach *et al.* 2008; Schmalenbach *et al.* 2009; Schmalenbach and Pillen 2009; Wang *et al.* 2010b). In addition, we are currently investigating variation in the S42ILs for traits like mineral nutrient content, nitrogen deficiency tolerance, drought tolerance and others. Based on the Illumina genotypes, we selected a core set of 32 S42ILs which represents the minimum number of lines required to achieve the *Hsp* genome coverage of the complete set. Such a core set is beneficial for an initial low-resolution genome-wide QTL screening, whereas additional lines carrying smaller introgressions can be used subsequently for QTL fine-mapping (Fridman *et al.* 2004; Keurentjes *et al.* 2007). The present set of S42ILs includes two large gaps of *Hsp* introgressions on chromosomes 5H and 3H. We assume that these gaps occurred by chance in our S42IL set due to the limited number of backcross lines we started with. We neither conducted a selection of phenotypes nor did we observe a reduced viability of plants during IL development. To achieve a complete donor genome coverage, we are currently selecting lines from BC_3_ and BC_4_ progenies of a pool of potential S42ILs.

The present and other studies demonstrate the usefulness of the Illumina GoldenGate assay for developing precisely characterized advanced backcross populations (Sato and Takeda 2009; Druka *et al.* 2011). Because barley genome sequencing is still in progress (Schulte *et al.* 2009), these populations are proposed as a key tool for high-resolution mapping of QTL and Mendelian loci and subsequent identification of the causal genes.

### Development of high-resolution mapping populations

The development of a library of HR populations for most of the original S42ILs will foster both map-based cloning and the transfer of interesting exotic genes that exhibit strong and/or favorable effects in the elite barley background. By means of the new HR populations, map-based cloning of QTL seems feasible since the available SNP data of S42ILs and the corresponding QTL data for pathogen resistances, yield-related traits and malting quality traits (Schmalenbach *et al.* 2008; Schmalenbach *et al.* 2009; Schmalenbach and Pillen 2009) can be used to directly select the appropriate S42IL-HR population from the HR library. The available number of up to 52,000 seeds for each HR population can thus be used to rapidly select recombination events in close vicinity to the target gene, allowing the genetic separation of tightly linked markers/genes from the target gene.

### Localization of the thresh-1 locus and identification of candidate genes

Within the S42IL population we identified the *thresh-1* locus on the long arm of chromosome 1H, where only line S42IL-143 carried the *Hsp* allele at the *thresh-1* locus. The *Hsp thresh-1* homozygous phenotype showed increased rachis and awn strength, which prevented them from breaking during normal mechanical threshing. To our knowledge, this is the first published report of such a phenotype in barley. The most well-studied grain threshing trait in barley is the brittle-rachis phenotype, where the grain spikes shatter upon applying slight mechanical force. The brittle-rachis phenotype is controlled by two tightly linked genes, *Btr1* and *Btr2*, located on chromosome 3H (Takahashi and Hayashi 1964). Therefore, based on the phenotypic and genetic differences, we concluded that *thresh-1* and *Btr1*/*Btr2* are unrelated.

During domestication of barley, the *Btr1*/*Btr2* genes were selected against by early farmers to prevent seed loss before harvesting. Similar findings hold true for wheat domestication where, over time, early farmers selected at independent loci the alleles *br*, *tg*, *sog*, and *Q* to control brittleness, glume tenacity, glume softness, and threshability of wheat spikes (Sood *et al.* 2009). It is likely that early farmers also selected against the *thresh-1* allele to facilitate easier harvesting. It remains open what evolutionary advantage the exotic *thresh-1* allele provided prior to domestication. The remaining of the awns or parts thereof with the grain might have increased the chances of long distance seed dispersal through animals. After map-based isolation of the *thresh-1* allele, further studies analyzing its allele frequency in wild barley populations might help to better understand the evolutionary history of this gene.

We were rapidly able to reduce the *thresh-1* locus to an interval of 4.3 cM by utilizing the S42IL-143HR high resolution mapping population, in combination with genotyping of polymorphic BOPA1 SNP markers for the target region. Furthermore, we could take advantage of the recent low-coverage sequencing of barley chromosome 1H and subsequent gene order prediction (Mayer *et al.* 2009) to identify candidate genes located in the *thresh-1* interval. Based on the increased mechanical force required to remove the rachis and awns from the seeds we hypothesize that the *thresh-1* gene is involved in altering the cell wall composition of the spike. In support of this hypothesis, the genomic *thresh-1* region is predicted to contain at least two orthologous cell wall related genes from rice encoding for cellulose synthase-like family C (CSLC7) and polygalacturonase proteins. In barley, the CSLC gene family is known to comprise at least four members (CSLC1-4) with CSLC1 being orthologous to the rice CSLC7 gene (Dwivany *et al.* 2009). The CSLC1 has been mapped in barley to the long arm of chromosome 1H in the vicinity of the *thresh-1* locus (Burton *et al.* 2010). The proposed function of the CSLC gene family in plants is to synthesize xyloglucan (Cocuron *et al.* 2007), which is a major component in strengthening cell walls. In contrast, polygalacturonase enzymes are involved in the degradation of pectin and subsequent weakening of the cell wall. In *Arabidopsis*, loss of function mutants for the polygalacturonase genes ADPG1 and ADPG2 caused the siliques not to shatter at maturity by preventing cells of the dehiscence zone from breaking apart (Ogawa *et al.* 2009). Based on this evidence, both CSCL1 and polygalacturonase are potential candidates for *thresh-1*. Further work is now under way to screen several thousand lines from the S42IL-143HR population to positionally clone and then functionally analyze the *thresh-1* gene.

## Supplementary Material

Supporting Information
