# Supplementary material for: High-Resolution Genotyping of Wild Barley Introgression Lines and Fine-Mapping of the Threshability Locus thresh-1 Using the Illumina GoldenGate Assay
Source: G3 (Bethesda). 2011 Aug 1;1(3):187–96. doi: 10.1534/g3.111.000182 (PMC3276139; doi:10.1534/g3.111.000182)
Supplement: Supporting Information [file supp_1_3_187__index.html]

Supporting Information 

# High-Resolution Genotyping of Wild Barley Introgression Lines and Fine-Mapping of the Threshability Locus *thresh-1* Using the Illumina GoldenGate Assay

## Supporting Information for Schmalenbach *et al.*, 2011

**Files in this Data Supplement:**

- Table S1 - Graphical genotypes of 73 S42ILs with 636 BOPA1 SNPs (Microsoft Excel, .xls, 288 KB)
- Table S2 - Methods and primers used to convert 32 BOPA Illumina markers into CAPS or pyrosequencing markers (Microsoft Excel, .xls, 64 KB)
- Table S3 - Predicted genes in the thresh-1 region between markers 1.0357 and HvABAIP based on the chromosome 1H virtual gene order (modified from Mayer et al. 2009) (Microsoft Excel, .xls, 20 KB)
